# Supplementary material for: Modulation of Lipid Metabolism by Trans-Anethole in Hepatocytes
Source: Molecules. 2020 Oct 26;25(21):4946. doi: 10.3390/molecules25214946 (PMC7662808; doi:10.3390/molecules25214946)
Supplement: Supplementary file 1 [file molecules-25-04946-s001.pdf]

Table 1. PCR primer sequences.

Name	Forward	Reverse	Size
CD36	GCAACAAACCACACACTGGG	AGACTGTGTTGTCCTCAGCG	736
FABP1	GCAAGTACCAACTGCAGAGC	AGTGCTTCCCATTCTGCACG	129
ApoB100	TTTCTGAGTCCCAGTGCCCA	TTAATGTGTATGAAGGCACCAGG	664
ApoC3	CCCGGGTACTCCTTGTTGTT	CCTCAGGGTCCAAATCCCAG	264
CPT1	CCACAGTCTCGCAAGGATGG	GCAAGTGGGGAGTTCTGGAG	399
GLUT2	CACTATAGACATGTTTTGGGTGTT	CCCATCAAGAGAGCTCCAAC	308
SREBP1c	AGTTTCCGAGGAACTTTTCGC	GCCGACTTCACCTTCGATGT	271

Figure S1. Expression levels for modulating genes of lipid metabolism in HepG2 cells .

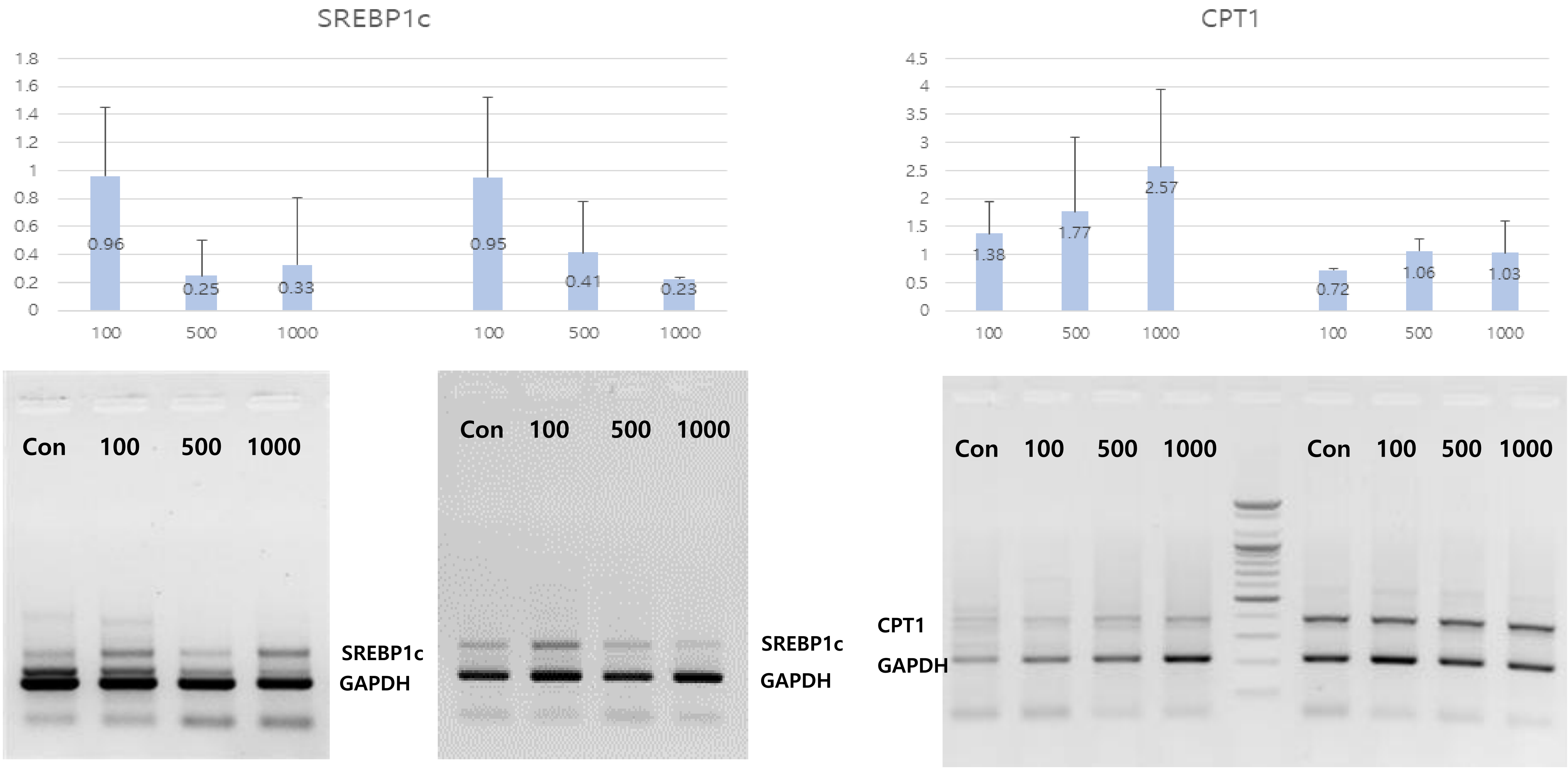

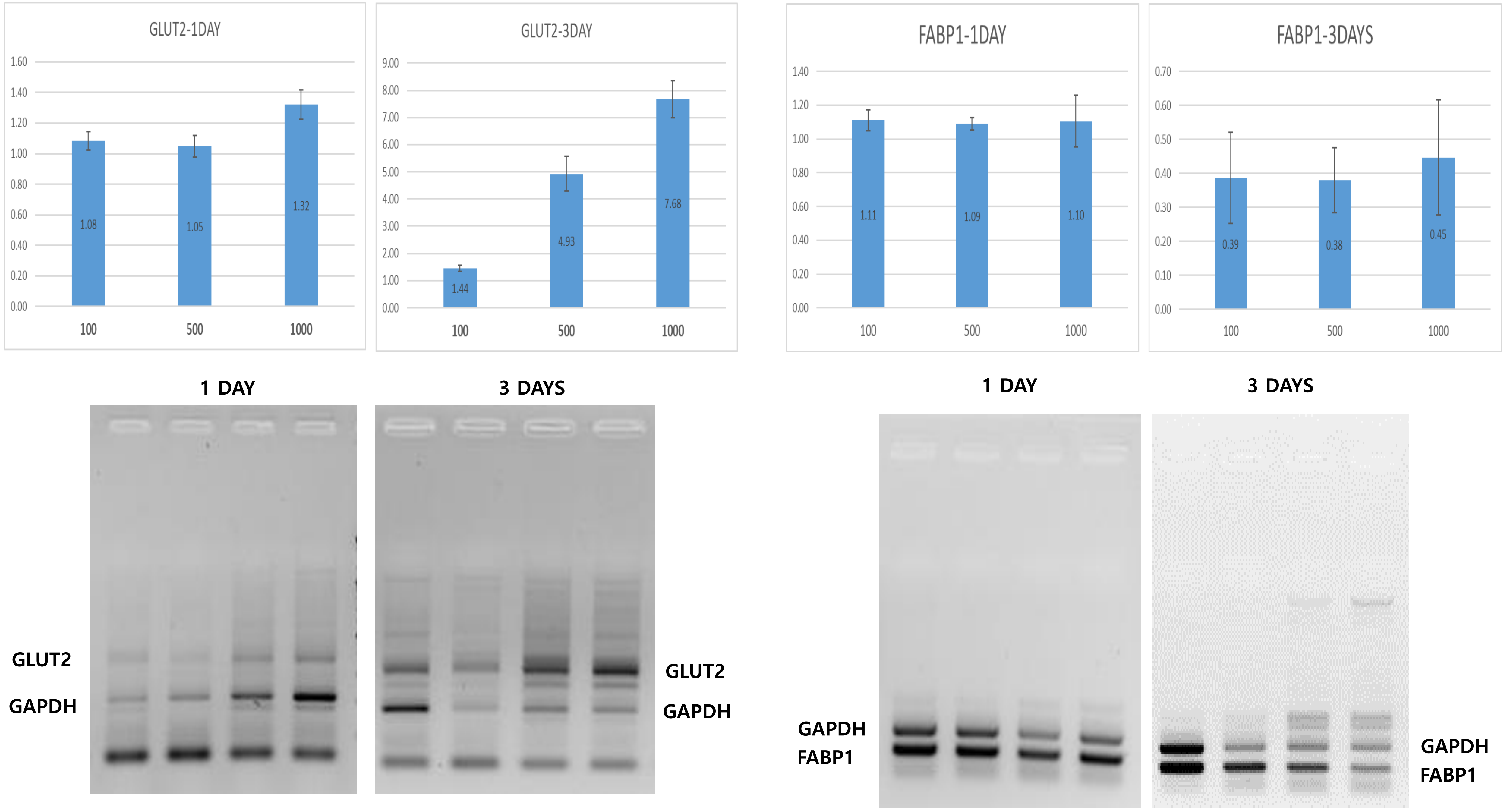
GLUT2-1DAY
1.60
1.40
1.20
1.00
0.80
0.60
0.40
0.20
0.00
1.08
1.05
1.32
100
500
1000
GLUT2-3DAY
9.00
8.00
7.00
6.00
5.00
4.00
3.00
2.00
1.00
0.00
1.44
4.93
7.68
100
500
1000
FABP1-1DAY
1.40
1.20
1.00
0.80
0.60
0.40
0.20
0.00
1.11
1.09
1.10
100
500
1000
FABP1-3DAYS
0.70
0.60
0.50
0.40
0.30
0.20
0.10
0.00
0.39
0.38
0.45
100
500
1000
1 DAY
3 DAYS
GLUT2
GAPDH
GLUT2
GAPDH
1 DAY
3 DAYS
GAPDH
FABP1
GAPDH
FABP1

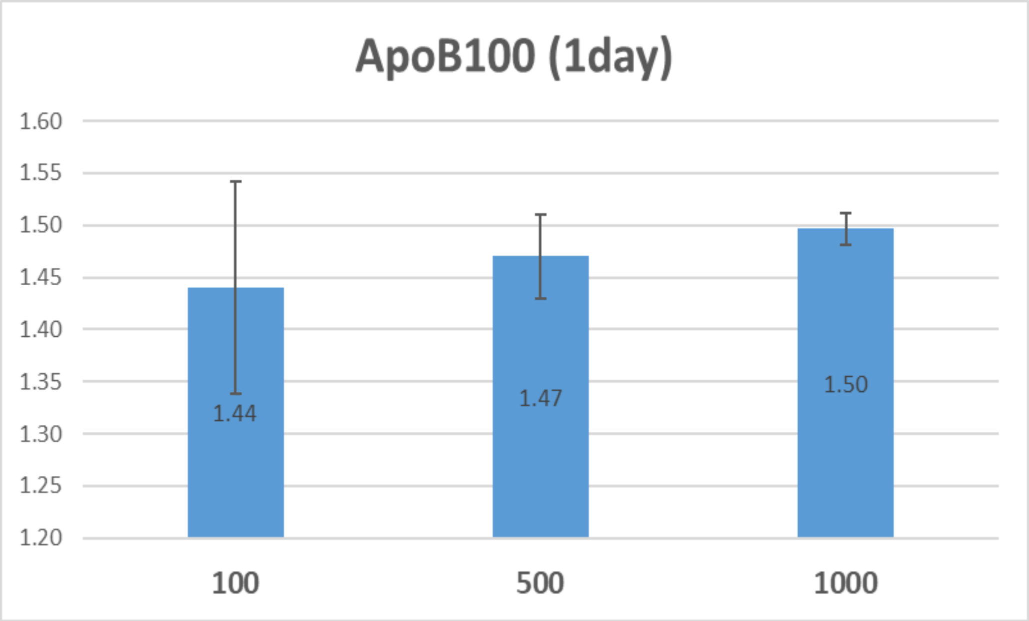
ApoB100 (1day)
1.60
1.55
1.50
1.45
1.40
1.35
1.30
1.25
1.20
1.44
1.47
1.50
100
500
1000

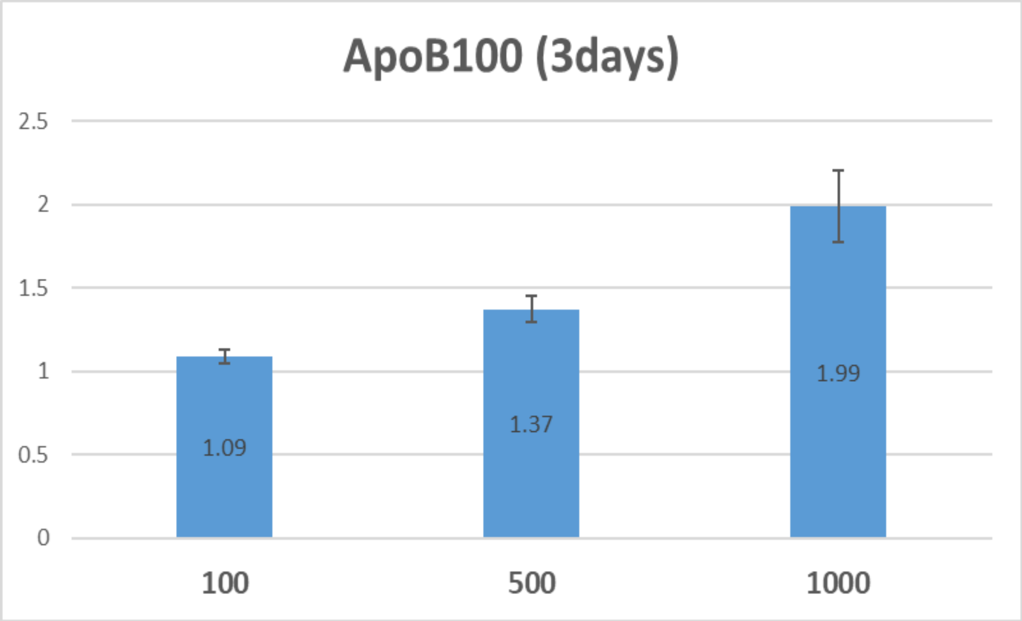
ApoB100 (3days)
2.5
2
1.5
1
0.5
0
1.09
1.37
1.99
100
500
1000

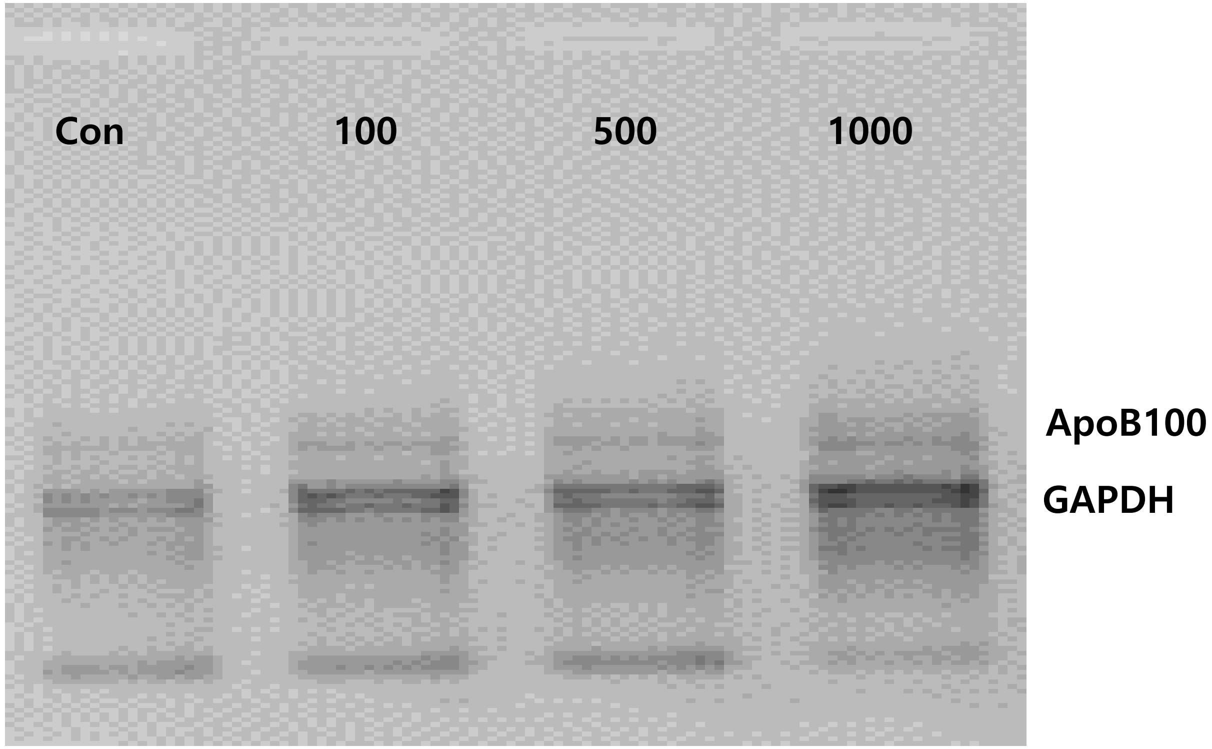
Con
100
500
1000
ApoB100
GAPDH

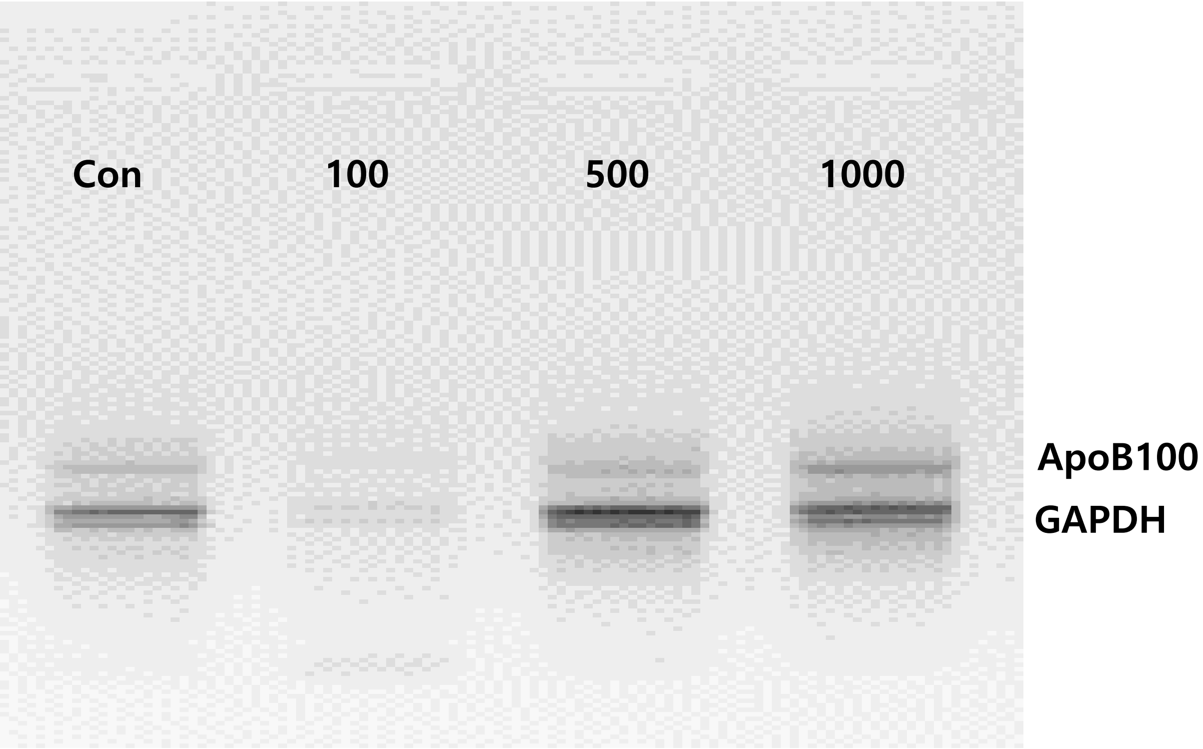
Con
100
500
1000
ApoB100
GAPDH

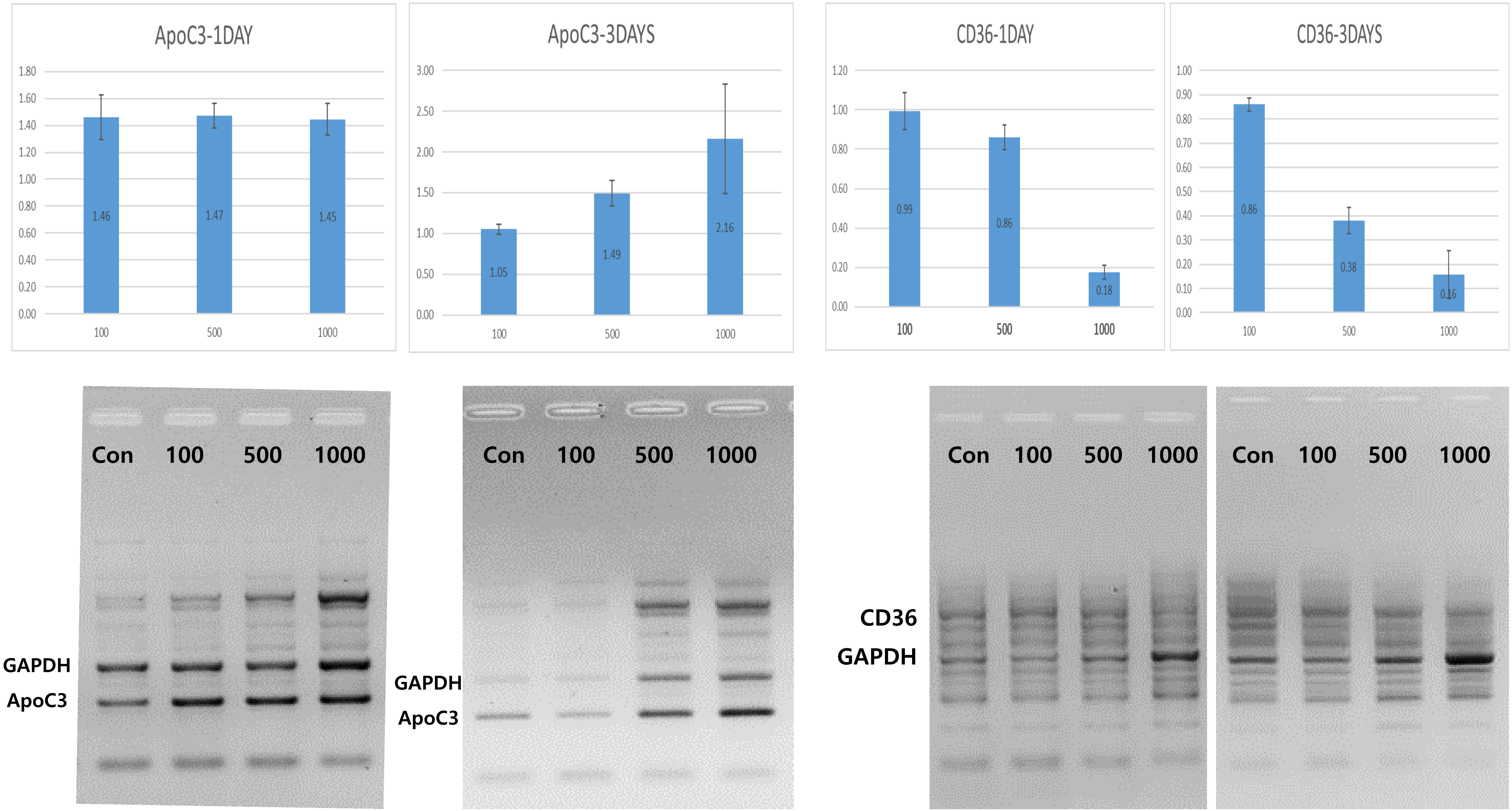
ApoC3-1DAY
1.80
1.60
1.40
1.20
1.00
0.80
0.60
0.40
0.20
0.00
1.46
1.47
1.45
100
500
1000
ApoC3-3DAYS
3.00
2.50
2.00
1.50
1.00
0.50
0.00
1.05
1.49
2.16
100
500
1000
CD36-1DAY
1.20
1.00
0.80
0.60
0.40
0.20
0.00
0.99
0.86
0.18
100
500
1000
CD36-3DAYS
1.00
0.90
0.80
0.70
0.60
0.50
0.40
0.30
0.20
0.10
0.00
0.86
0.38
0.16
100
500
1000
Con 100 500 1000
GAPDH
ApoC3
Con 100 500 1000
GAPDH
ApoC3
Con 100 500 1000
CD36
GAPDH
Con 100 500 1000
